# Supplementary material for: Splice-Junction-Based Mapping of Alternative Isoforms in the Human Proteome
Source: Cell Rep. Author manuscript; Available in PMC 2020 Jan 15. (PMC6961840; doi:10.1016/j.celrep.2019.11.026)
Supplement: 2 [file NIHMS1546469-supplement-2.zip › DF1/PXD006675/LeftAtrium/LeftAtrium_22_TTN_RVEAEPAEVTEFR.pdf]

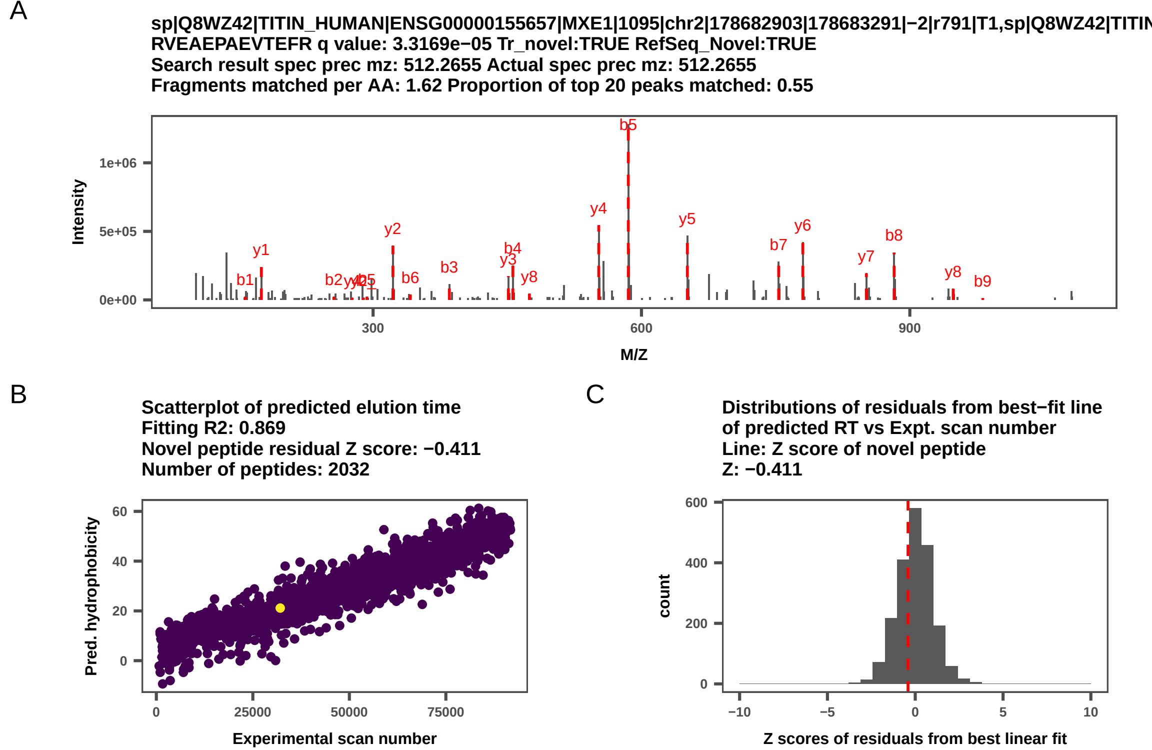
A
sp|Q8WZ42|TITIN_HUMAN|ENSG00000155657|MXE1|1095|chr2|178682903|178683291|−2|r791|T1,sp|Q8WZ42|TITIN
RVEAEPAEVTEFR q value: 3.3169e−05 Tr_novel:TRUE RefSeq_Novel:TRUE
Search result spec prec mz: 512.2655 Actual spec prec mz: 512.2655
Fragments matched per AA: 1.62 Proportion of top 20 peaks matched: 0.55
Intensity
M/Z
b5
y4
y5
y6
b8
b7
y2
y1
b4
y3
y8
b3
b6
b1
b2
y7
y8
b9
B
Scatterplot of predicted elution time
Fitting R2: 0.869
Novel peptide residual Z score: −0.411
Number of peptides: 2032
Pred. hydrophobicity
Experimental scan number
C
Distributions of residuals from best−fit line
of predicted RT vs Expt. scan number
Line: Z score of novel peptide
Z: −0.411
count
Z scores of residuals from best linear fit
